# Antiherpetic Activity of Taurisolo^®^, a Grape Pomace Polyphenolic Extract

**DOI:** 10.3390/microorganisms11051346

**Published:** 2023-05-20

**Authors:** Carla Zannella, Annalisa Chianese, Giuseppe Annunziata, Annalisa Ambrosino, Anna De Filippis, Gian Carlo Tenore, Ettore Novellino, Mariano Stornaiuolo, Massimiliano Galdiero

**Affiliations:** 1 Department of Experimental Medicine, University of Campania “Luigi Vanvitelli”, 80138 Naples, Italy; carla.zannella@unicampania.it (C.Z.); annalisa.chianese@unicampania.it (A.C.); annalisa.ambro92@libero.it (A.A.); anna.defilippis@unicampania.it (A.D.F.); massimiliano.galdiero@unicampania.it (M.G.); 2 Department of Pharmacy, University of Naples Federico II, 80131 Naples, Italy; giuseppe.annunziata@unina.it (G.A.); giancarlo.tenore@unina.it (G.C.T.); 3 Department of Medicine and Surgery, Università Cattolica del Sacro Cuore, 00168 Rome, Italy; ettore.novellino@unicatt.it

**Keywords:** natural compounds, antiviral activity, Taurisolo^®^, polyphenols, wine extracts, herpes

## Abstract

Herpes simplex virus (HSV) is widespread in the population, causing oral or genital ulcers and, rarely, severe complications such as encephalitis, keratitis, and neonatal herpes. Current available anti-HSV drugs are acyclovir and its derivatives, although long-term therapy with these agents can lead to drug resistance. Thus, the discovery of novel antiherpetic compounds merits additional studies. In recent decades, much scientific effort has been invested in the discovery of new synthetic or natural compounds with promising antiviral properties. In our study, we tested the antiviral potential of a novel polyphenol-based nutraceutical formulation (named Taurisolo^®^) consisting of a water polyphenol extract of grape pomace. The evaluation of the antiviral activity was carried out by using HSV-1 and HSV-2 in plaque assay experiments to understand the mechanism of action of the extract. Results were confirmed by real-time PCR, transmission electron microscope (TEM), and fluorescence microscope. Taurisolo^®^ was able to block the viral infection by acting on cells when added together with the virus and also when the virus was pretreated with the extract, demonstrating an inhibitory activity directed to the early phases of HSV-1 and HSV-2 infection. Altogether, these data evidence for the first time the potential use of Taurisolo^®^ as a topical formulation for both preventing and healing herpes lesions.

## 1. Introduction

Viral infections are becoming more frequent and pose a threat to public health. The research and identification of new antiviral molecules are necessary to guarantee new therapeutic options as antiviral drugs become less effective due to the emergence of resistant viral strains. Herpes simplex virus (HSV) is a member of *Herpesviridae*, a wide family of enveloped-DNA viruses able to cause clinically significant syndromes in both adults and neonates [1,2]. HSV types 1 and 2 cause the most common viral skin infections; therefore, many scientists test natural products to evaluate their anti-HSV effects in reducing the infectivity of the virus both in vivo and in vitro [3,4,5]. Diffusion of these viruses is very high, about 80% for HSV-1, with a recurrence of 40%. Herpesviruses can persist throughout the lifetime of the host; then, after the primary lytic infection, the virus hides in the nerve cells where it establishes a latent infection until viral reactivation. After primary infection of epithelial cells, the virus becomes latent in neurons of the peripheral nervous system and reactivates with recurrent episodes [6]. Reactivation of latent HSV, especially during the deficiency of immunity, induces recurrent infections and transmission to new hosts. Usually, antiviral agents acting on viral adsorption, inhibition of viral penetration into cells, and inhibition of viral release, are used to treat herpes viral infection. The most common antiviral drugs used in clinical practice are nucleoside or nucleotide analogs affecting the DNA replication process, including acyclovir, penciclovir, valacyclovir, famciclovir, ganciclovir, and cidofovir [7]. In addition, non-nucleoside or nucleotide drugs can also be used to treat herpetic infection. In such a way, Foscarnet interacts with the viral DNA-polymerase blocking nucleotides from binding and incorporation into the DNA strand. However, in immunocompromised patients, prolonged treatment with these drugs is more likely to develop drug-resistant strains.

Natural products have always been an inexhaustible source of new antiviral drugs [8,9]. Many plant extracts have inhibitory activity in the replication of many viruses [10]. Due to their multifunctional components, plant extracts have also demonstrated broad-spectrum activity against drug-resistant viruses [11]. Their mechanism of action is focused on the viral life cycle, including viral entry, fusion, replication, assembly, and specific interactions between virus–host [12]. Studies conducted with natural molecules such as resveratrol have demonstrated the possibility of inhibiting HSV infection in vivo and in vitro. When added within 1 h of in vitro infection, resveratrol has potent anti-HSV activity by activating NF-κB within the nucleus in Vero cells and modulating the expression of essential genes in immediate–early, early and late, and viral DNA synthesis [13]. Studies conducted on mouse models have shown that the topical use of different compositions of resveratrol significantly inhibits the development of skin lesions induced by HSV-1, limiting erythema and desquamation.

Among the food-derived bioactive compounds, polyphenols emerge for their potential as antiviral agents with activity against various pathogens such as enterovirus 71 [14], Epstein-Barr virus [15,16], influenza virus [17], and other viruses responsible for respiratory infections [18,19,20], including the pandemic severe acute respiratory syndrome coronavirus 2 (SARS-CoV-2) [21]. This evidence suggests the utility to further investigate the effect of polyphenols, whose antioxidant and anti-inflammatory potential are historically known. Polyphenols are ubiquitously present in a huge variety of plants and, by extension, in foods that are commonly used for polyphenol-based nutraceutical formulations. Among these, grapes are a valid source of polyphenols, mainly represented by resveratrol, procyanidins, and catechins [22,23,24]. Interestingly, previous studies investigated the antiviral potential of grape pomace extracts against a number of human pathogens, including HSV [Grape Canes from Typical Cultivars of Campania (Southern Italy) as a Source of High-Value Bioactive Compounds: Phenolic Profile, Antioxidant and Antimicrobial Activities], hepatitis A [25] and C [26] viruses, SARS-CoV-2 [27], human enteric virus [28], and human immunodeficiency virus type 1 [29].

In this study, we evaluated the antiviral activity of Taurisolo^®^ (a novel nutraceutical formulation based on grape pomace polyphenolic extract) against herpetic infection, showing a significant reduction in the viral infection with its activity directed at the viral particle in the extracellular and early phases of viral infection. In detail, we selected two members belonging to the *Herpesviridae* family (HSV-1 and HSV-2). Both viruses are responsible for frequent infections in the world. They present a different range of infections; in detail, HSV-1 is mainly correlated to oral herpes transmitted by oral-to-oral contact, while HSV-2 is principally responsible for genital herpes transmitted sexually.

## 2. Materials and Methods

### 2.1. Nutraceutical Formulation

In this study, cells were treated with a novel polyphenol-based nutraceutical formulation (registered as Taurisolo^®^) consisting of an Aglianico cultivar grape polyphenolic extract. An initial pilot formulation was provided by the Department of Pharmacy, University of Naples Federico II, Naples, Italy. Subsequently, MB-Med Company (Turin, Italy) manufactured the large-scale production. For the Taurisolo^®^ production, as well as polyphenol composition, please see Supplementary Materials.

### 2.2. Cell and Virus Culture

Vero cells (ATCC CCL-81, Manassas, VA, USA) were grown in Dulbecco’s Modified Eagle Medium (DMEM) with 4.5 g/L glucose (Microtech, Naples, Italy) supplemented with antibiotic solution 100× (100 IU/mL penicillin and 100 μg/mL streptomycin; Himedia, Naples, Italy), and 10% Fetal Bovine Serum (FBS, Microtech). HSV-1 (strain SC16), containing a lacZ gene driven by the cytomegalovirus IE-1 promoter to express β-galactosidase, HSV-2 (strain G, ATCC VR-734), and fluorescent HSV-1, containing the GFP reporter inserted into the gene coding for the VP22 tegument protein [30], were propagated on Vero cells, as previously reported [31]. As an unenveloped virus, Enterovirus C (Sb-1, poliovirus Sabin strain chat, ATCC VR-1562) was cultured on the Vero cell line.

### 2.3. Cytotoxicity

Vero cells were seeded in 96-well plates (2 × 10^4^ cells/mL) and incubated for 24 h at 37 °C in 5% CO_2_. The cytotoxicity was evaluated by the 3-(4,5-Dimethylthiazol-2-yl)-2,5-Diphenyltetrazolium Bromide (MTT, Sigma-Aldrich, St. Louis, MO, USA) assay. The cells were incubated with several concentrations of Taurisolo^®^ (from 800 to 0.12 µg/mL); then, after 2 and 24 h, all wells were treated with 100 μL of MTT solution (5 mg/mL) and incubated at 37 °C for 3 h. Subsequently, 100 μL of DMSO 100% (Sigma-Aldrich) was added to each well to dissolve the formazan salts for 10 min with vigorous agitation at room temperature (RT), and absorbance was measured at 570 nm. A total of 100 μL DMSO 100% was used for negative control (ctr−), while 100 μL of culture medium represented the positive control (ctr+). The viability of cells was evaluated compared to the control cells.

### 2.4. Antiviral Activity

The antiviral activity of the Taurisolo^®^ and maltodextrins was evaluated through plaque assays. Vero cells were seeded in 24-well plates (1 × 10^5^) and incubated overnight at 37 °C. Four treatment assays were performed, as previously described (co-treatment, virus pretreatment, cell pretreatment, and post-treatment assays) [32]. Noncytotoxic concentrations of Taurisolo^®^ and maltodextrins were tested in all assays and infected with the virus at a multiplicity of infection (MOI) of 0.01 PFU/mL (plaque forming units per milliliter). After the time of infection, for each treatment, cells were washed with citrate buffer (pH 3), overlaid with DMEM supplemented with carboxymethylcellulose (CMC, Sigma, C5678, C5013) 5% for 48 h, and fixed and stained with 4% formaldehyde and 0.5% crystal violet. Plaques were counted and the percentage of viral inhibition was calculated in relation to the nontreated control (ctr−). Two additional assays were performed to understand viral attachment and entry. In the former, the cell monolayer was infected with HSV-1 at MOI 0.01 and treated with compounds simultaneously for 1 h at 4 °C. In the entry assay, cells were infected with the virus for 1 h at 4 °C and after were treated with Taurisolo^®^ for 1 h.

### 2.5. Evaluation of Viral Gene Expression

Vero cells were seeded in the same conditions as previously reported. The virus pretreatment assay was performed in the range from 800 to 0.12 µg/mL. The RNA genome was collected by TRIzol reagent (Thermo Fisher Scientific, Waltham, MA, USA) after 48 h and quantified through the absorbance at NanoDrop (NanoDrop 2000, Thermo-Fisher Scientific). RNA was retrotranscribed to cDNA by 5× All-In-One RT Master Mix (Applied Biological Materials, Richmond, VA, Canada), and through a quantitative polymerase chain reaction, cDNA was amplified. The expression of specific viral genes, UL54 (immediate early gene), UL52 (early gene), and UL27 (late gene), was evaluated. The relative target threshold cycle (Ct) values were normalized using a housekeeping gene, the glyceraldehyde 3-phosphate dehydrogenase (GAPDH). Finally, the mRNA levels were calculated using the 2-ΔΔCt method. All the synthetic and experimental details are summarized in Table 1. Oligos were provided by Eurofins (Ebersberg, Germany).

### 2.6. Virus Purification and Morphological Analysis by TEM

HSV-1 was purified by density gradient ultracentrifugation with cesium chloride (CsCl). Transmission electron microscopy (TEM) analysis was performed using an FEI TECNAI G2 S-twin 200 kV apparatus operating at 120 kV (LaB6 source) microscope. Taurisolo^®^ (at 25 μg/mL) and purified virus were incubated for 1 h at 37 °C. Next, 10 µL of the mixture was transferred onto a formvar/carbon TEM grid (Merck, Readington Township, NJ, US) and then left at RT until complete evaporation of the solvent. Finally, the sample was stained using 2% phosphotungstic acid (pH 6.5) for 30 s to obtain the contrast.

### 2.7. Statistical Analysis

All tests were performed in triplicate and indicated as mean ± standard deviation (SD) evaluated by GraphPad Prism (version 8.0.1). One-way ANOVA followed by Dunnett’s multiple comparisons test was used; a value of *p* ≤ 0.05 was considered significant.

## 3. Results

### 3.1. Cytotoxicity

The effect of Taurisolo^®^ and maltodextrins was evaluated on cell viability by incubating VERO cells with different concentrations of each compound for 2 and 24 h (2 h for resembling the time of compound incubation on cells and 24 h used as a long exposure indicator). Cytotoxicity activity was analyzed by the MTT assay. Different concentrations of compounds were tested in the range from 800 to 0.012 µg/mL. The cell viability was expressed as a percentage of viability compared to the non-treated cells. No toxicity was observed at all concentrations tested (Supplementary Material, Table S1). Only Taurisolo^®^ at the highest tested concentration (800 µg/mL), showed 40% of toxicity (Supplementary Table S1).

### 3.2. Antiviral Activity

The antiviral activity of Taurisolo^®^ and maltodextrins was investigated against different types of viruses. To better understand the mechanism of action, we performed several in vitro plaque assays against enveloped and non-enveloped viruses.

In brief, all noncytotoxic concentrations were examined in four schemes of treatment in which what changed was the time of addition of the compound. In the co-treatment assay, the cell monolayer was treated simultaneously with compounds and infected with the virus. To evaluate the interaction of Taurisolo^®^ with the cellular or viral surface, the cells or virus were first pretreated with Taurisolo^®^, in cell pretreatment or virus pretreatment assay. Finally, a post-treatment assay was carried out to evaluate the interference of the compound with intracellular targets or phases of infection. In this case, the cells were infected with the virus and then treated with the compound.

**Figure 1:**
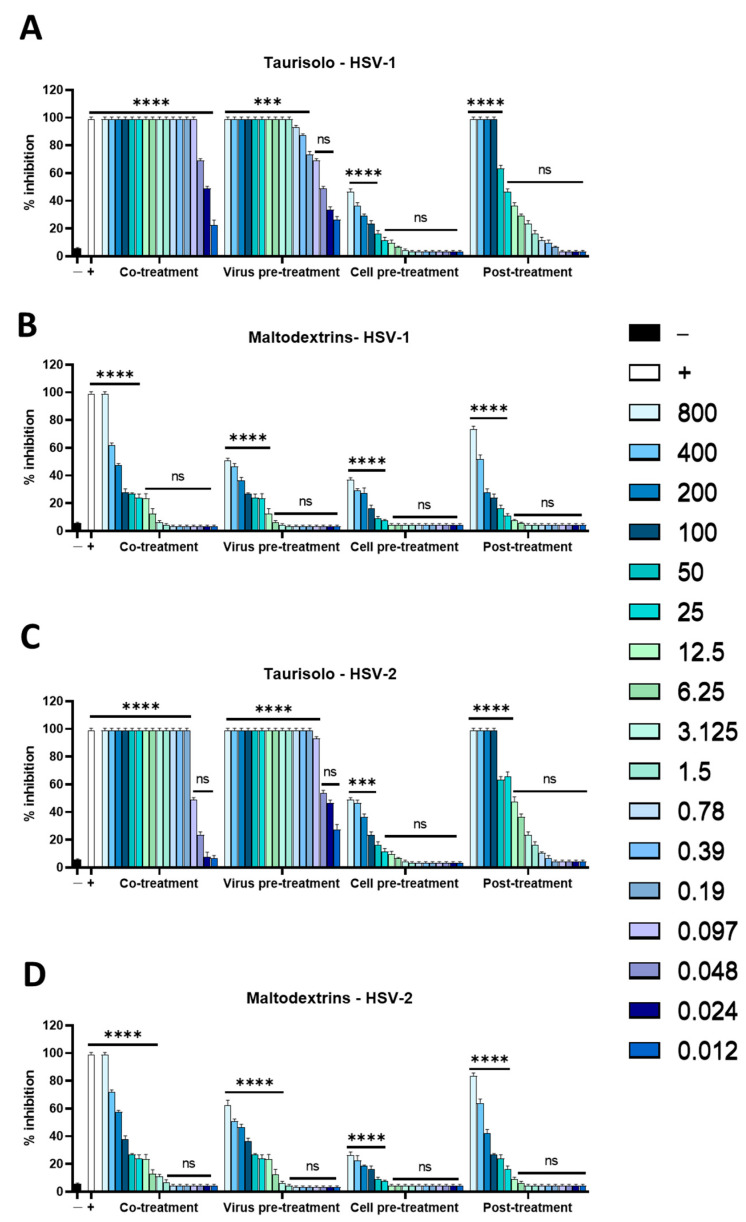
Antiviral activity against HSV-1 and HSV-2. Different assays were performed to evaluate antiherpetic activity: (**A**) Taurisolo^®^ against HSV1; (**B**) maltodextrins against HSV-1; (**C**) Taurisolo^®^ against HSV-2; (**D**) maltodextrins against HSV-2. **** *p* < 0.0001; *** *p* < 0.0001; ns: nonsignificant.

In the first test, carried out to understand if the compound had an inhibitory action, the cells were treated with Taurisolo^®^ and simultaneously infected with the viruses (co-treatment). As reported in Figure 1A,C, the compound showed 50% inhibition at the concentrations of 0.024 and 0.0975 µg/mL against HSV-1 and HSV-2, respectively. To exclude that this action could be due to maltodextrins, plaque assays were carried out in the same conditions previously described. We observed antiviral potential of maltodextrins, confirming that Taurisolo^®^ was endowed with an antiherpetic effect (Figure 1B,D).

Furthermore, to better investigate which phase of the viral life cycle the extract was able to interfere with, additional tests were executed. The virus pretreatment and cell pretreatment tests indicated whether the extract could act in the extracellular phase of infection, directly with the virus or with the cell membrane, respectively. Figure 1A,C showed the high inhibitory effect of Taurisolo^®^ in the virus pretreatment assay, with an IC_50_ of 0.097 and 0.024 µg/mL for HSV-1 and HSV-2, respectively. Comparatively, a reduction in infection was observed in the cell pretreatment assay indicating that Taurisolo^®^ did not interact with the cell surface, but rather with the viral envelope.

Finally, the post-treatment suggested whether the extract could prevent intracellular stages of infection. A moderate inhibition of viral infection was detected, particularly at concentrations of 50 and 25 µg /mL for both viruses (Figure 1A,C).

**Figure 2:**
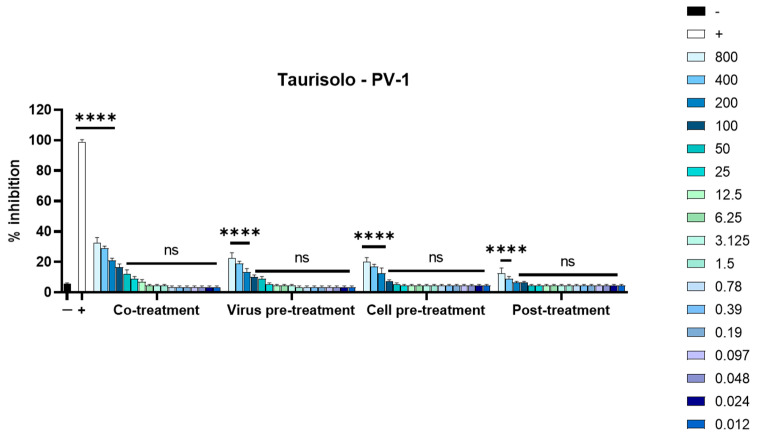
Antiviral activity against PV-1. Co-treatment, virus pretreatment, cell pretreatment, and post-treatment assays were performed to evaluate the inhibitory power of Taurisolo^®^ against poliovirus type 1. **** *p* < 0.0001; ns: nonsignificant.

The inhibitory activity of Taurisolo^®^ was further investigated against a nonenveloped viral model. Poliovirus type 1 (PV-1), an enterovirus with a single-stranded RNA genome, was used. Plaque assays were carried out under the same conditions. The data shown in Figure 2 demonstrate that the extract did not reduce the infection, evidencing that it was able to interact with the viral lipid membrane.

### 3.3. Molecular Analysis

#### Evaluation of Viral Gene Expression

**Figure 3:**
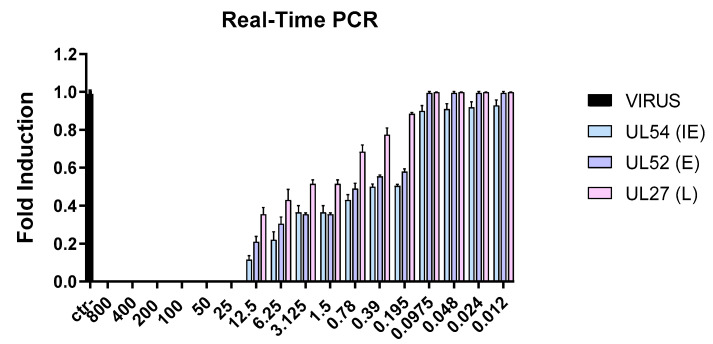
Molecular assay. Real-time PCR was performed to evaluate the effect of Taurisolo^®^ on viral gene expression. For HSV-1, the expression of immediate early (UL54), early (UL52), and late (UL27) genes were analyzed. Ctr− refers to infected but not treated cells.

To confirm the data obtained in vitro by plaque assays, the Taurisolo^®^ antiviral effect was analyzed by quantifying the expression levels of genes involved in the viral infection. In detail, the expression of three viral genes involved in the HSV infection was analyzed: (i) the immediate early gene UL54, coding for the ICP27 protein; (ii) the early gene UL52, coding for the DNA primase; and (iii) the late gene UL27, coding for the structural glycoprotein B (gB) [33,34]. First, a virus pretreatment assay was carried out, RNA was collected after 24 h, converted to cDNA, and then amplified by real-time PCR. Results showed that the extract interfered with viral replication by blocking the expression of viral genes. Data showed that the infection was reduced in a dose-dependent manner, demonstrating the virucidal potential of Taurisolo^®^. As reported in Figure 3, by decreasing the Taurisolo^®^ concentration, viral gene expression increased and reached the same level of virus control from 0.0975 to 0.012 µg/mL. 

### 3.4. Microscopy Analyses

The antiviral effect of Taurisolo^®^ was also analyzed by using microscope analyses by fluorescence and electron microscopy.

#### 3.4.1. HSV-1 GFP

**Figure 4:**
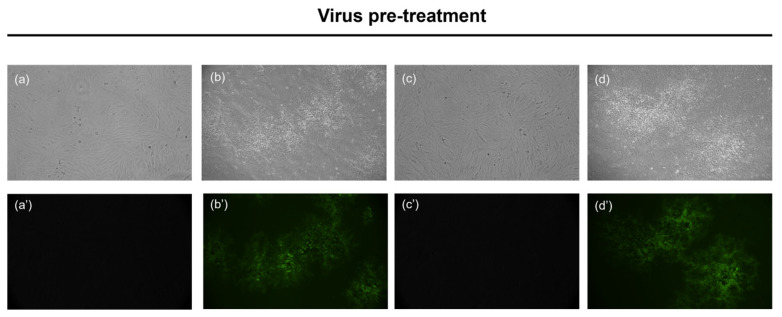
Antiviral activity against HSV-1-GFP. Images (**a**,**a′**) show uninfected and untreated cells; (**b**,**b′**) represent infected cells. No plaques were observed when cells were stimulated with the compound at 0.78 µg/mL in (**c**,**c′**). While the images (**d**,**d′**) show plaques in cells treated with 0.012 µg/mL. Magnificence: 20×.

Fluorescence microscopy analysis was performed by infecting the cell monolayer with the engineered virus HSV-1-GFP, in which the green fluorescent protein (GFP) had been inserted into the genome, in the gene encoding the tegument protein VP22 [31]. A virus pretreatment assay was conducted under the same conditions previously described, and the fluorescence was recorded 48 h postinfection. Figure 4 shows the bright field (RGB) images in the upper panel and GFP images in the lower panel.

As shown in Figure 4, at a concentration of 0.78 µg/mL, in which Taurisolo^®^ inhibited the viral infection, no fluorescence signal was detected; on the contrary, the fluorescence signal was high at nonactive concentrations (0.012 µg/mL). The fluorescence microscopy data confirmed the results of the plaque reduction test.

#### 3.4.2. TEM

**Figure 5:**
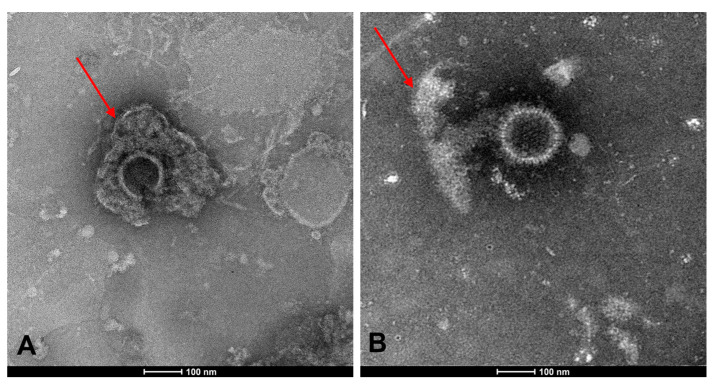
TEM observation of HSV-1 after treatment with Taurisolo^®^. (**A**) Control HSV-1 (10^9^ PFU/mL) with its complete spherical structure and the external envelope, indicated by the red arrow. (**B**) HSV-1 treated with Taurisolo**^®^** showed complete destruction of the viral envelope (indicated by the red arrow). Negative staining treatment with a phosphotungstic acid solution (2%) was performed. Magnificence: 100 nm.

To visualize the effect of Taurisolo^®^ on the viral envelope, HSV-1 particles were treated with the compound and observed by transmission electron microscope (TEM). Briefly, the purified virus was incubated with Taurisolo^®^ at the inhibitory concentration of 25 μg/mL and subsequently placed on carbon-coated copper grids, as described in the Section 2. Figure 5 demonstrates that Taurisolo^®^ could act on the viral envelope by blocking the herpetic infection.

## 4. Discussion

In the present study, we demonstrated the effect of a novel nutraceutical formulation based on grape pomace polyphenolic extract containing Taurisolo^®^ in inhibiting herpetic infection. From the literature, we know that grape extracts are endowed with antimicrobial activity against various microorganisms. This activity is due to a higher number of polyphenols found in grapes skin, seed, shoot, and stem [35,36,37,38,39,40,41]. However, none of these studies have described a possible antiviral effect. Recently, our group analyzed the antimicrobial potential of grape wine extracts from three different cultivars of Campania, Italy, i.e., Aglianico, Fiano, and Greco [42]. First, we found that an alkaline pH was able to yield the best polyphenol-rich extracts. The Aglianico extract exhibited a mild antibacterial effect only against *Staphylococcus aureus*, probably due to its peculiar cell wall composition, very different from that of Gram-negative bacteria, surrounded by an outer membrane of lipopolysaccharide. The most representative result was that observed against viruses. Very strong antiviral activity was noted for all the extracts against the herpetic infection; in particular, the Greco extract was the most active to interact directly with the viral particles until the low dose of 10 μg/mL, by inhibiting both HSV-1 and HSV-2 initial stages of infection. We have also shown the potential of *Vitis vinifera* leaf extract against SARS-CoV-2 [27] and, even in this case, the extract exhibited a virucidal effect on the viral envelope, probably by destroying the viral surface layer and preventing the subsequential fusion event with the target cells.

Since the early step in the viral lifecycle is viral attachment onto the host cells, interfering with viral attachment and entry would prevent viral infection. To date, few entry inhibitors have been approved as antiviral drugs by the Food and Drug Administration (FDA) [43]. One of them is docosanol, an anti-HSV drug approved in 2000 as the only clinical medication for cold sores and fever blisters. It is also active against other viruses such as the respiratory syncytial virus (RSV) and other herpesviruses (varicella zoster virus and cytomegalovirus) [44,45,46] since its action is directed to membrane phospholipids. Docosanol can also be used in synergism with other anti-HSV drugs [45] and it works well against acyclovir-resistant HSV [47].

Currently, HSV infections are among the most widespread viral infections. From the last available report of 2016, 67% of the world population was affected by HSV-1, and 13% by HSV-2. Alongside these huge numbers, antiviral compounds are scarce, and more strains continue to acquire resistance to traditional drugs.

Natural compounds could be an effective alternative to fight viral infections. The biological activity of Taurisolo^®^ has been widely investigated [48,49,50,51,52,53,54,55,56,57]. In the last year, a phase II multicentric clinical trial (TAEROVID-19) has been performed to test the safety and feasibility of Taurisolo^®^ aerosol formulation in the treatment of COVID-19 patients [58]. A rapid reduction in symptoms and in hospitalizations in intensive care was observed. These positive outcomes could be due to the well-known anti-inflammatory and antioxidant effects of Taurisolo^®^. In this context, we reported for the first time the antiherpetic activity of Taurisolo^®^. We observed a strong reduction in the viral infectivity of both HSV-1 and HSV-2. In detail, the inhibitory activity was mainly directed at the viral particles, as shown by IC_50_ at very low concentrations of 0.097 and 0.024 μg/mL, respectively, for HSV-1 and HSV-2 (Figure 1). This result was further validated when Taurisolo^®^ was incubated with a nonenveloped virus of PV-1 and antiviral activity was detected (Figure 2). The virucidal activity of the extract was also confirmed by analyzing the expression level of some viral genes involved in HSV-1 replication (Figure 3). All genes were not expressed until 25 μg/mL of Taurisolo^®^. Then, we observed a dose-dependent increase especially in late gene expression once the extract concentration decreased. These data suggested that the extract possessed an inhibitory action in the early stages of infection when the virus was required to attach and enter the host cells. Most likely, Taurisolo^®^ covered the viral particles and destroyed them, as demonstrated by TEM images (Figure 5), preventing the next phases of infection. To date, the antiherpetic activity of Taurisolo^®^ has not been investigated; even though most natural extracts containing a very similar profile of polyphenols have already reported antiviral effects against herpesvirus infection. *Melissa officinalis L.* is a mixture of ferulic acid, caffeic acid, and rosmarinic acid, with the ability to inhibit HSV-1 infection after 6 h of treatment [59]. *Ficus carica* contains caffeic acid, 3,4-dihydrobenzoic acid, p-OH-phenylacetic acid, p-coumaric acid, luteolin, N-arginine, and ferulic acid, with a virucidal activity when incubated with HSV-1 for 1 h at 78 μg/mL [60]. Propolis extract is a complex mixture consisting of aromatic acids including E-p-coumaric acid, benzoic acid, ferulic acid, fatty acids, flavonoids, and sugars, able to interfere with both HSV-1 and HSV-2 particles at very early stages of infection, i.e., adsorption in host cells [61]. All these examples clearly show that the antiviral effect derives from a mixture of polyphenols and not from individual components.

Finally, our results indicate a strong effect of Taurisolo^®^ extract in preventing herpetic infections caused by HSV-1 and HSV-2.

## 5. Conclusions

In summary, we analyzed the effects of the natural extract Taurisolo^®^ against two members of the *Herpesviridae* family. We found that this compound can inhibit herpetic infection through direct nonspecific interactions with viral surface components. On the contrary, it was inactive against the nonenveloped poliovirus. We hypothesized that Taurisolo^®^ could act on the viral envelope as a virucidal agent, suggesting a potential use as a topical treatment for both preventing and healing herpes lesions.

## Figures and Tables

**Table 1:** Oligos sequences for viral genes and thermal conditions used for real-time PCR.

Gene Symbol	Forward Sequence	Reverse Sequence
HSV-1 UL54	TGGCGGACATTAAGGACATTG	TGGCCGTCAACTCGCAG
HSV-1 UL52	GACCGACGGGTGCGTTATT	GAAGGAGTCGCCATTTAGCC
HSV-1 UL27	GCCTTCTTCGCCTTTCGC	GCCTTCTTCGCCTTTCGC
GAPDH	CCTTTCATTGAGCTCCAT	CGTACATGGGAGCGTC
Thermocycler condition		
95 °C for 10 min $\begin{array}{c} 95\;{}^{\circ}C\;\mathrm{for}\;15\;s\;\\ 60\;{}^{\circ}C\;\mathrm{for}\;1\;\min\\ 72\;{}^{\circ}C\;\mathrm{for}\;20\;s \end{array}\}$ (40 cycles)		

## Data Availability

The data presented in this study are available on request from the corresponding author. The authors can confirm that all relevant data are included in the article.

## Supplementary Materials

The following supporting information can be downloaded at: https://www.mdpi.com/article/10.3390/microorganisms11051346/s1, Figure S1: Chromatogram of Taurisolo^®^ with peaks relative to polyphenol fraction. Table S1: Cytotoxic activity of Taurisolo^®^ and Maltodextrins evaluated by MTT assay. Different concentrations of compounds were tested (800–0.012 µg/mL) and data were expressed as percentage of viability.
