# Correction: Zannella et al. Antiherpetic Activity of Taurisolo^®^, a Grape Pomace Polyphenolic Extract. *Microorganisms* 2023, *11*, 1346

**DOI:** 10.3390/microorganisms13051117

**Published:** 2025-05-13

**Authors:** Carla Zannella, Annalisa Chianese, Giuseppe Annunziata, Annalisa Ambrosino, Anna De Filippis, Gian Carlo Tenore, Ettore Novellino, Mariano Stornaiuolo, Massimiliano Galdiero

**Affiliations:** 1 Department of Experimental Medicine, University of Campania “Luigi Vanvitelli”, 80138 Naples, Italy; carla.zannella@unicampania.it (C.Z.); annalisa.chianese@unicampania.it (A.C.); annalisa.ambro92@libero.it (A.A.); anna.defilippis@unicampania.it (A.D.F.); massimiliano.galdiero@unicampania.it (M.G.); 2 Department of Pharmacy, University of Naples Federico II, 80131 Naples, Italy; giuseppe.annunziata@unina.it (G.A.); giancarlo.tenore@unina.it (G.C.T.); 3 Department of Medicine and Surgery, Università Cattolica del Sacro Cuore, 00168 Rome, Italy; ettore.novellino@unicatt.it

In the original publication [1], there was a mistake in “Figure 4. Antiviral activity against HSV-1-GFP” as published. In Figure 4, two panels (a and c) were wrongly assembled. The error happened by confusing the two panels representing untreated cells (a) and cells that were not affected by the treatment (c). The corrected Figure 4 appears below. The authors state that the scientific conclusions are unaffected.


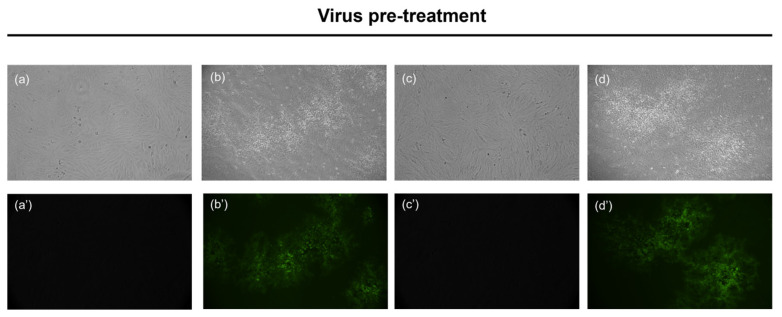


This correction was approved by the Academic Editor. The original publication has also been updated.
