# Supplementary material for: Antiherpetic Activity of Taurisolo®, a Grape Pomace Polyphenolic Extract
Source: Microorganisms. 2023 May 20;11(5):1346. doi: 10.3390/microorganisms11051346 (PMC10222725; doi:10.3390/microorganisms11051346)
Supplement: Supplementary file 1 [file microorganisms-11-01346-s001.zip › microorganisms-2380645-supplementary.pdf]

Supplementary Material

Methods

Taurisolo® formulation

To obtain the Taurisolo® briefly, grapes were extracted with hot water (50°C), and the solution was filtrated and concentrated and underwent a spray-drying process with maltodextrins as support (5–15%) obtaining a fine microencapsulated powder that was analyzed by High-Performance Liquid Chromatography-diodearray detector (HPLC-DAD, Jasco Inc., Easton, MD, USA). The Taurisolo® polyphenol profile is the following: Ferulic acid 14.59 ± 0.98 µg/g, Resveratrol 12.55 ± 0.02 µg/g, Caffeic acid 35.00 ± 3.00 µg/g, p-coumaric acid 122.75 ± 2.77 µg/g, Rutin 98.81 ± 7.31 µg/g, Quercetin 135.41 ± 4.69 µg/g, Procyanidin B1 dimer 946.33 ± 55.20 µg/g, Procyanidin B2 dimer 645.89 ± 59.17 µg/g, Syringic acid 310.95 ± 0.01 µg/g, Epicatechin 1696.55 ± 109.60 µg/g, Gallic acid 199.46 ± 4.59 µg/g; Catechin 2499.04 ± 307.41 µg/g.

UHPLC-UV profile (280-330 nm) of Taurisolo® via ESI-IT-TOF-MS/MS

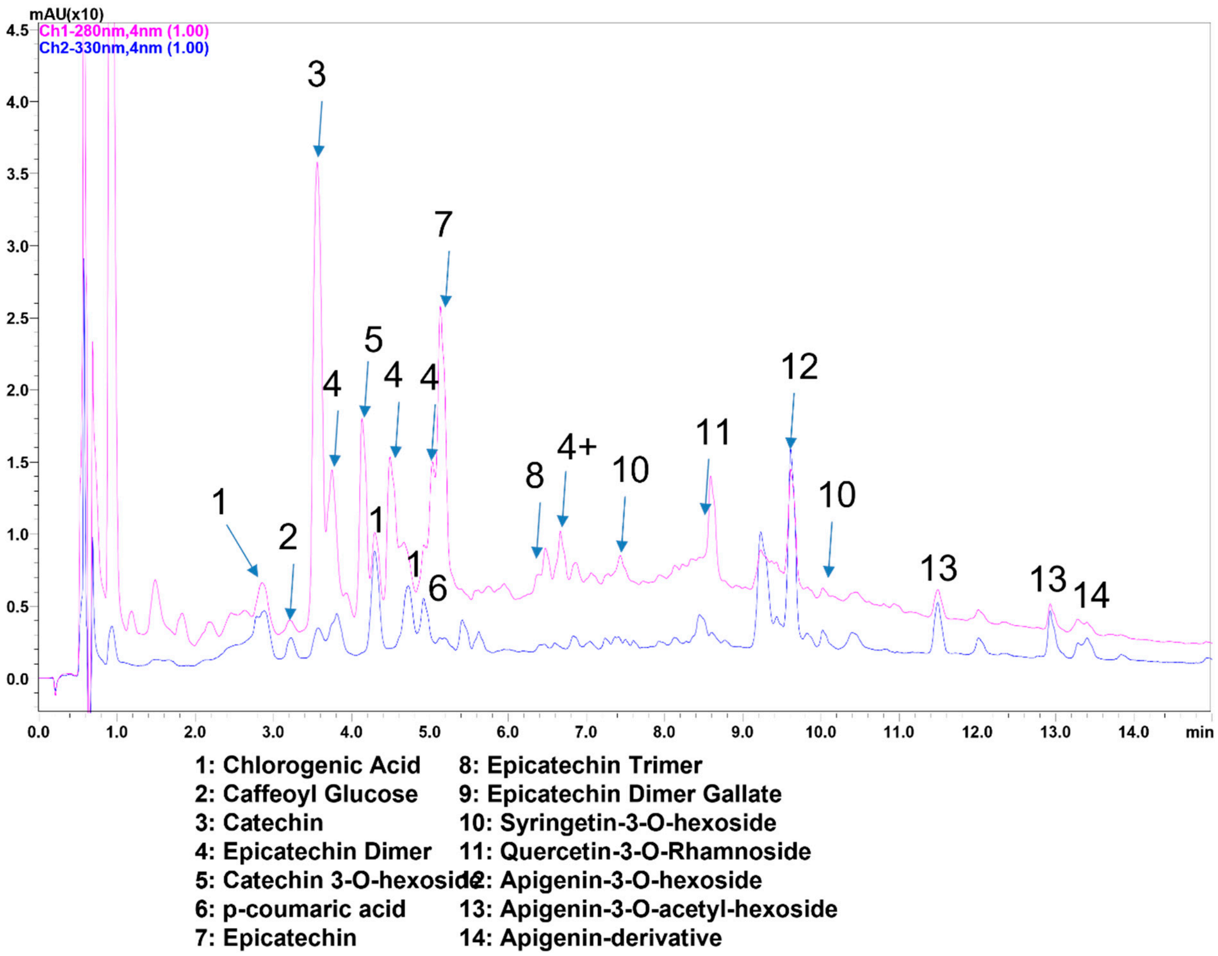

Figure S1. Chromatogram of Taurisolo® with peaks relative to polyphenol fraction.

Results*Cytotoxicity*

	Taurisolo®	Maltodextrins
[μg/mL]	% cell viability	
800	40	98.5
400	88.05	99.24
200	98.48	100
100	100	100
50	100	100
25	100	100
12.5	100	100
6.25	100	100
3.125	100	100
1.5	100	100
0.78	100	100
0.39	100	100
0.19	100	100
0.097	100	100
0.048	100	100
0.024	100	100
0.012	100	100

Table S1. Cytotoxic activity of Taurisolo® and Maltodextrins evaluated by MTT assay. Different concentrations of compounds were tested (800-0.012μg/mL) and data were expressed as percentage of viability.
